# Between-year vocal aging in female red deer (*Cervus elaphus*)

**DOI:** 10.1186/s13104-018-3833-4

**Published:** 2018-10-17

**Authors:** Ilya A. Volodin, Olga V. Sibiryakova, Nina A. Vasilieva, Elena V. Volodina, Vera A. Matrosova, Andrés J. Garcia, Francisco J. Pérez-Barbería, Laureano Gallego, Tomás Landete-Castillejos

**Affiliations:** 1 Department of Vertebrate Zoology, Faculty of Biology, Lomonosov Moscow State University, Vorobievy Gory, 12/1, Moscow, 119991 Russia; 2 Scientific Research Department, Moscow Zoo, B. Gruzinskaya, 1, Moscow, 123242 Russia; 3 Severtsov Institute of Ecology and Evolution RAS, Leninskii pr. 33, Moscow, 119071 Russia; 4 Engelhardt Institute of Molecular Biology RAS, Vavilov str., 32, Moscow, 119991 Russia; 5 Animal Science Tech, Applied to Wildlife Research Group. IREC (UCLM-CSIC-JCCM), and Sec. Rec. Cinegéticos, Instituto de Desarrollo Regional, Universidad de Castilla-La Mancha, 02071 Albacete, Spain

**Keywords:** Mammal voice, Non-human senescence, Ungulate, Female red deer, *Cervus elaphus*, Nasal and oral contact calls, Body weight and condition, Welfare, Social discomfort measure

## Abstract

**Objectives:**

Studying animal vocal aging has potential implication in the field of animal welfare and for modeling human voice aging. The objective was to examine, using a repeated measures approach, the between-year changes of weight, social discomfort score (bites of other hinds on hind pelt), body condition score (fat reserves) and acoustic variables of the nasal (closed-mouth) and the oral (open-mouth) contact calls produced by farmed red deer hinds (*Cervus elaphus*) toward their young.

**Results:**

Repeated measures ANOVA revealed that with an increase of hind age for 1 year, the acoustic variables of their nasal contact calls (the beginning and maximum fundamental frequencies, the depth of frequency modulation and the peak frequency) decreased, whereas in their oral contact calls only the end fundamental frequency decreased. Duration and power quartiles did not change in any call type. Body weight and body condition score increased between years, whereas discomfort score decreased. Results of this study revealed directly the short-term effects of aging on the acoustics of the nasal contact calls in the same hinds. This study also confirmed that elevated emotional arousal during emission of the oral contact masks the effects of aging on vocalization in female red deer.

**Electronic supplementary material:**

The online version of this article (10.1186/s13104-018-3833-4) contains supplementary material, which is available to authorized users.

## Introduction

Studies of vocal aging have potential welfare implications in both nonhuman mammals [1, 2] and humans [3, 4]. Comparison of voices in female red deer (*Cervus elaphus*) aged from 4 to 18 years revealed that fundamental frequency (f0) of hind contact calls decreases with age but increases with degree of social discomfort [2]. The effects of aging differed on hind nasal and oral contact calls: a greater number of variables related to f0 decreased in the lower-arousal nasal calls compared to only one (the maximum fundamental frequency, f0max) in the high-arousal oral calls [2]. The shift of f0 toward higher frequencies with increase of emotional arousal is a widespread phenomenon in mammals [5–10] that in red deer hinds masks the effect of aging on vocal variables [2].

However, the findings that both aging and social discomfort have measurable effects on the acoustic properties of a red deer female’s nasal and oral contact calls [2] were not yet confirmed with longitudinal (year-to-year) investigation. Longitudinal studies of vocal aging are relatively rare for humans [4, 11–13] and nonhuman mammals [14] because of difficulties with collection of representative data for long terms [15]. So far, a single longitudinal study of vocal changes with aging in nonhuman mammals is only available for fallow deer *Dama dama* bucks [14]. In this short-term longitudinal study we use a repeated measures approach to show directly how different variables change in the same animals with an increase of age for 1 year. The particular aim of this study was to examine the between-year changes of weight, social discomfort score (bites of other hinds on hind pelt), body condition score (fat reserves) and acoustic variables of the nasal (closed-mouth) and the oral (open-mouth) contact calls produced by farmed Iberian red deer hinds toward their young.

## Main text

### Methods

Calls of individually marked red deer hinds were recorded from 10.06.2011 to 27.06.2011 and from 14.06.2012 to 23.06.2012 at the experimental farm of the University of Castilla-La Mancha (Albacete, Spain) in frames of previous studies [16, 17]. At this captive population, the age of hind first pregnancy is usually 16–17 months of age, and the age of last pregnancy is usually 18 years, however there were a few cases observed when some hinds had calves at ages of 20–21 years. The longevity is usually about 19 years, although some hinds reached up to 22 years.

All hinds in both years of recording were kept together with their calves younger 1 month of age in permanent groups (4 groups in 2011 and 3 groups in 2012) separately from adult stags and yearlings. The hinds vocalized toward their calves when separated by a distance over 10 m for different reasons, either inside or outside the outdoor enclosures. The animals kept visual contact and desired to join but something prevented the joining (e.g. a wire-mesh fence or fear of researcher standing in between the mother and calf) [16].

We collected 30 h of audio recordings (16 h in 2011 and 14 h in 2012) from the 13 hinds from which recordings were available in both years. Age of these 13 hinds recorded in both years ranged from 4 to 13 years (mean ± SD = 10.31 ± 2.84) in 2011 and from 5 to 14 years (11.31 ± 2.84) in 2012 (see Additional file 1 for details).

Methods of acoustic recordings followed the studies [2, 16], conducted on the same herd. For acoustic recordings (48 kHz, 16 bit) we used solid state recorders Marantz PMD-660 (D&M Professional, Kanagawa, Japan) with Sennheiser K6-ME66 cardioid electret condenser microphones (Sennheiser electronic, Wedemark, Germany). The distance from the hand-held microphone to the animals was between 5 and 35 m; the level of recording was adjusted during the recordings accordingly to the intensity of the produced calls. We recorded calls daily for 28 days in total (18 days in 2011 and for 10 days in 2012) from 6:00–7:00 to 12:00–13:00, often with synchronous video for documenting the oral or nasal vocal emission, using a digital camcorder Panasonic HDC-HS100 (Panasonic Corp., Kadoma, Japan). During recordings, individual identities of callers producing calls through the mouth and through the nose were labeled by voice [2, 16].

All animals were weighed with Mettler-Toledo ID1 scales (Mettler-Toledo S.A.E., Barcelona, Spain) as the part of routine farm management [18] one time during the periods of acoustic recordings in each year. All animals were scored for body condition score and for discomfort score (Additional file 1 and see [2] for details).

The condition score represented a standard body condition index, varying from 1 to 5, scored from 1 = emaciated to 5 = obese [2, 19, 20]. The discomfort score was a proxy of the number of bites on the pelt of the animal, thus representing an index related to being recipient of social aggression described in detail by [2] for this study herd. Such aggression is similar to those reported in the wild in reindeer *Rangifer tarandus* feeding in small snow craters [21, 22]. Score 1 = all the hair of the deer is intact. Score 2 = occasional lack of hair, mainly in the sides and rear quarters. Less than 10% naked (bald) skin. Score 3 = substantial lack of hair on sides and rear quarters. Less than one-third of the skin naked. Score 4 = substantial lack of hair on sides, rear quarters and also in neck. Less than two-thirds of naked skin. Score 5 = lack of hair very substantial. Less than 10% of the skin with hair considering neck, sides and rear quarters including upper part of the four legs, from elbow/knew upwards [2]. The discomfort score is inverse to hind social rank: in our study population, the older is the hind, the lower is its discomfort score and therefore the higher is its social rank [2].

For acoustic analyses, following the studies [16, 17] we only used individually identified calls of known call type (nasal or oral), not disrupted by wind, overlapped by calls of other animals or saturated with very high amplitude in the recording. To avoid pseudoreplication, we took calls from different recording sessions per animal and from different parts within session, because calls from the same sequence are commonly more similar in their acoustic structure than calls from different sequences [23].

From the 13 hinds only 7 provided both oral and nasal calls, whereas the remaining 6 hinds only provided the nasal calls. Thus, for further acoustic analyses and calculating the average values of acoustic variables per individual hind, we took from 1 to 26 (on average 13.23 ± 5.96) high-quality nasal calls per individual per year from 13 hinds and from 3 to 25 (on average 11.57 ± 5.85) high-quality oral calls per individual per year from 7 hinds. Two individuals provided only one nasal call. In total, we analyzed 531 calls (187 oral and 344 nasal).

Acoustic analyses were conducted in the same way for the oral and nasal calls. For each nasal and oral call, we measured the same set of nine acoustic variables following [2, 16]. We measured: duration, start (f0beg), maximum (f0max) and end (f0end) fundamental frequencies and the fpeak, representing the frequency of maximum amplitude and the q25, q50 and q75, representing the lower, medium and upper quartiles, covering 25, 50 and 75% of the energy of the call spectrum respectively.

**Fig. 1:**
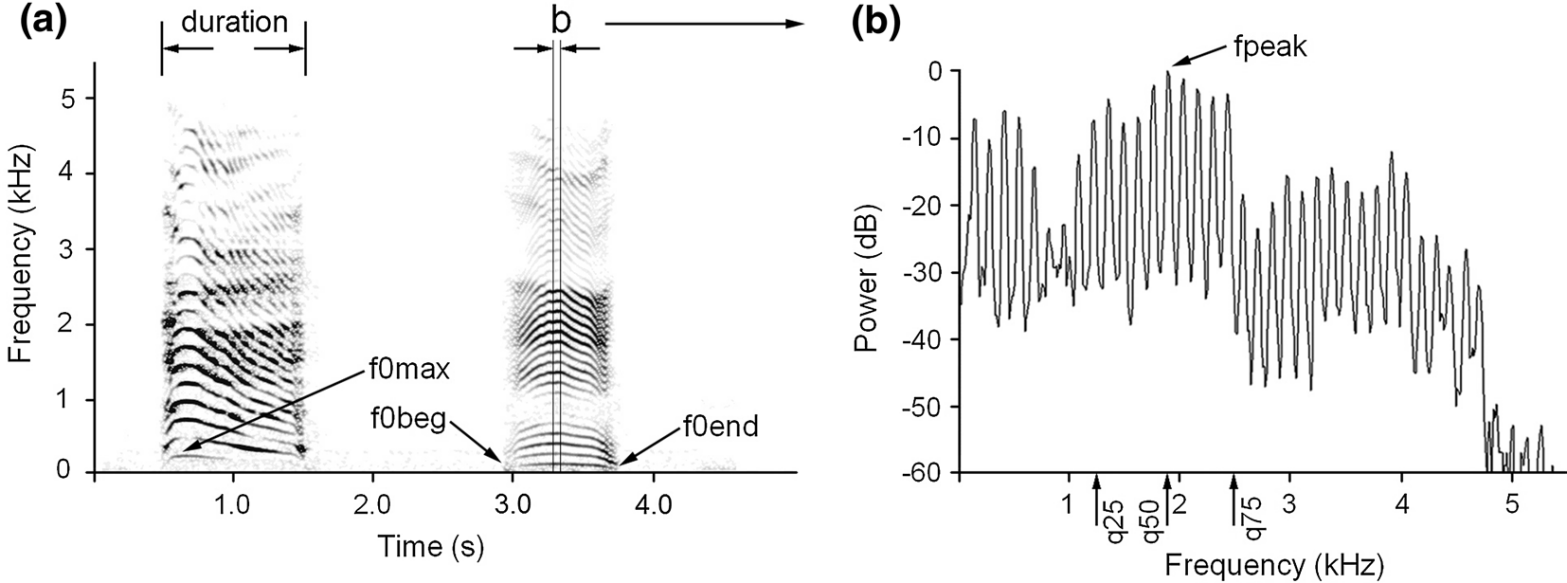
Measured acoustic variables. **a** Spectrogram of hind oral (left) and nasal (right) calls. **b** Mean power spectrum of 0.05 s fragment of a nasal call. Designations: duration—call duration; f0max—the maximum fundamental frequency; f0beg—the fundamental frequency at the onset of a call; f0end—the fundamental frequency at the end of a call; fpeak—the frequency of maximum amplitude within a call; q25, q50 q75—the lower, the medium and the upper quartiles, covering respectively 25, 50 and 75% energy of a call spectrum. The spectrogram was created with Hamming window; 11,025 kHz sampling rate; FFT 1024 points; frame 50%; and overlap 93.75%. Original wav-files are available in Additional file 2

Before measurements, the calls were downsampled to 11,025 Hz and high-pass filtered at 50 Hz to increase frequency resolution and to reduce the low-frequency background noise. We measured the duration of each call manually on the screen with the reticule cursor in the spectrogram window (Hamming window, FFT 1024 points, frame 50% and overlap 96.87%) by using Avisoft SASLab Pro software (Avisoft Bioacoustics, Berlin, Germany). Then, we performed manual measurements on the screen with the standard marker cursor of the f0beg, f0max and f0end of each call (Fig. 1). In a 0.05 s call fragment symmetrical about f0max (comprising about 5–10% of average call duration), we created the power spectrum, from which we automatically measured fpeak, q25, q50 and q75 (Fig. 1). Measurements were exported automatically to Microsoft Excel (Microsoft Corp., Redmond, WA, USA). In addition, for each call we selected the minimum f0 (f0min) as the minimum value between f0beg and f0end and calculated the depth of frequency modulation df0 as the difference between f0max and f0min. For subsequent acoustic analyses, we calculated the average values of acoustic variables per individual hind respectively for oral and nasal calls.

Statistical analyses were conducted using STATISTICA v. 13.0 (StatSoft, Tulsa, OK, USA). Means are given as mean ± SD, all tests were two-tailed, and differences were considered significant whenever *p *< 0.05. All dependent variables were normally distributed (Shapiro–Wilk W test, *p* > 0.05). We applied a repeated measures ANOVA to compare the mean values of acoustic variables, body weight, discomfort score and condition score between years.

### Results

Separately for samples of oral and nasal contact calls, we calculated means of mean values of acoustic variables for 2011 and for 2012 (Table 1). Repeated measures ANOVA showed, that as hinds age for 1 year, the values of the beginning fundamental frequency, the maximum fundamental frequency, the depth of frequency modulation and the peak frequency decrease in the nasal calls whereas only the values of the end fundamental frequency decrease in the oral calls (Table 1). The duration and power quartiles of the oral and nasal calls did not change between years. Repeated measures ANOVA also showed that as hinds age for 1 year, their body weight and condition score increase whereas the discomfort score decrease (Table 2).

**Table 1:** Values (mean ± SD) of oral and nasal hind call variables and repeated measures ANOVA results for comparison the mean values between 2011 and 2012 years separately for oral and nasal calls

Acoustic variable	Nasal calls (n = 13 hinds)			Oral calls (n = 7 hinds)		
	2011	2012	ANOVA	2011	2012	ANOVA
duration (s)	0.84 ± 0.29	0.81 ± 0.28	*F*_*1,12*_ = 0.16; *p *= 0.69	0.68 ± 0.21	0.68 ± 0.22	*F*_*1,6*_ = 0.03; *p *= 0.88
f0beg (Hz)	139 ± 23	127 ± 30	*F*_*1,12*_ = *8.41; p * = * 0.013*	141 ± 28	129 ± 16	*F*_*1,6*_ = 2.08; *p * = 0.20
f0max (Hz)	180 ± 31	173 ± 30	*F*_*1,12*_ = 5.21; *p * = 0.04	184 ± 27	178 ± 34	*F*_*1,6*_ = 0.53; *p * = 0.49
f0end (Hz)	88 ± 14	81 ± 21	*F*_*1,12*_ = 3.16; *p * = 0.10	88 ± 7	83 ± 7	*F*_*1,6*_ = 7.14; *p * = 0.037
df0 (Hz)	91 ± 26	78 ± 24	*F*_*1,12*_ = 15.82; *p * = 0.002	96 ± 26	82 ± 31	*F*_*1,6*_ = 2.12; *p * = 0.20
fpeak (Hz)	1076 ± 515	808 ± 601	*F*_*1,12*_ = 4.16; *p * = 0.06	1404 ± 308	1063 ± 308	*F*_*1,6*_ = 3.75; *p * = 0.10
q25 (Hz)	681 ± 186	667 ± 227	*F*_*1,12*_ = 0.07; *p * = 0.80	903 ± 218	887 ± 268	*F*_*1,6*_ = 0.03; *p * = 0.88
q50 (Hz)	1571 ± 261	1627 ± 282	*F*_*1,12*_ = 0.88; *p * = 0.37	1699 ± 123	1667 ± 261	*F*_*1,6*_ = 0.11; *p * = 0.76
q75 (Hz)	2526 ± 171	2739 ± 318	*F*_*1,12*_ = 4.09; *p *= 0.07	2532 ± 180	2616 ± 209	*F*_*1,6*_ = 1.21; *p * = 0.31

Designations: duration—call duration; f0beg—the fundamental frequency at the onset of a call; f0max—the maximum fundamental frequency of a call; f0end—the fundamental frequency at the end of a call; df0—the depth of frequency modulation, calculated as the difference between f0max and f0min; fpeak—the frequency of maximum amplitude within a call; q25, q50, q75—the lower, medium and upper quartiles of a call. Significant differences are highlighted in underlined

**Table 2:** Values (mean ± SD) of weight, discomfort score and condition score variables and repeated measures ANOVA results for comparison the mean values between 2011 and 2012 years

Variable	2011	2012	ANOVA
Weight (kg)	98.4 ± 10.4	105.6 ± 12.7	*F*_*1,12*_ = 20.49; *p* < 0.001
Discomfort score	1.87 ± 0.70	1.21 ± 0.22	*F*_*1,12*_ = 18.70; *p* < 0.001
Condition score	3.44 ± 0.23	3.94 ± 0.37	*F*_*1,12*_ = 24.00; *p* < 0.001

*n *= 13 hinds

Significant differences are highlighted in italic

### Discussion

This study indicates that the age-related shifts in voices can be detected even at terms as short as 1 year. In addition, these results confirm the data showing that nasal contact calls are better indicators of vocal aging than the oral contact calls, in female red deer [2]. Effects of aging are expressed in many variables of fundamental frequency of the nasal calls, produced by more relaxed hinds, compared with only one in the oral calls, produced by hinds at higher emotional arousal [2, 16]. This confirms the opposite effects on female mammal voices of aging on one side, and social discomfort and emotional arousal, on the other side [2]. The female aging results in decrease of voice fundamental frequency [2, 3, 24–28], whereas both emotional arousal and discomfort result in increase of fundamental frequency [2, 5, 6, 9, 10].

At the same time, the repeated measures approach applied in this study is not designed to capture the effects revealed in the preceding cross-sectional study [2]: effect of decrease of call duration, peak frequency and power quartiles with decrease of discomfort score, representing an index of being recipient of social aggression from other hinds. These effects are characteristic for mammalian callers [5, 6, 9, 10], including red deer [2, 17, 29] and fallow deer [7] and callers across other taxa of vertebrates [8, 30].

The short-term longitudinal approach, used previously for investigating effects of aging between years in wild fallow deer males [14], had proved its efficiency also for captive female cervids in this study. Other longitudinal studies considering the vocal changes with time in non-human animals were focused on the stable/recognizable vocal signature, as for fur seals [31], ground squirrels [32, 33], gazelles [34], cranes [35]; red deer [15, 16] and marmosets [36].

## Limitations

This study had two limitations:

- The study was conducted in one herd of farmed red deer, what limits expansion of results for other farmed or wild populations of red deer.
- Context of vocalizing (hind calling toward a calf) can only be used for hinds in reproductive age, not for *subadult* or *senex* age classes of female red deer.

## Additional files

**Additional file 1.** Table presenting age, weight, discomfort score and condition score of particular Iberian red deer hinds and mean acoustic measurements of their oral and nasal contact calls in 2011 and 2012.

**Additional file 2.** The Iberian red deer hind oral and nasal contact calls, recorded in context of communication with the calf.

## Abbreviations

f0beg: call beginning fundamental frequency
f0max: call maximum fundamental frequency
f0end: call end fundamental frequency
df0: the depth of frequency modulation
fpeak: call maximum amplitude frequency
q25: call lower power quartile
q50: call medium power quartile
q75: call upper power quartile
